# Distinct Characteristics of *Escherichia coli* Isolated from Patients with Urinary Tract Infections in a Medical Center at a Ten-Year Interval

**DOI:** 10.3390/pathogens10091156

**Published:** 2021-09-08

**Authors:** Wei-Hung Lin, Yen-Zhen Zhang, Po-Yao Liu, Po-Shun Chen, Shining Wang, Pei-Yun Kuo, Tran Thi Dieu Thuy, Tran Thi Thuy Duong, Li-Li Wen, Yi-Hsien Hsieh, Ming-Cheng Wang, Cheng-Yen Kao

**Affiliations:** 1 Institute of Clinical Medicine, College of Medicine, National Cheng Kung University, Tainan 701, Taiwan; dindonwhlin@hotmail.com; 2 Division of Nephrology, Department of Internal Medicine, National Cheng Kung University Hospital, College of Medicine, National Cheng Kung University, Tainan 701, Taiwan; 3 Institute of Microbiology and Immunology, School of Life Sciences, National Yang Ming Chiao Tung University, Taipei 112, Taiwan; yenjean0326@gmail.com (Y.-Z.Z.); eric50215@gmail.com (P.-Y.L.); i34044025@gs.ncku.edu.tw (S.W.); togebi87303@gmail.com (P.-Y.K.); dieuthuy28021997@gmail.com (T.T.D.T.); tranthithuyduong190198@gmail.com (T.T.T.D.); 4 Department of Biotechnology and Laboratory Science in Medicine, School of Biomedical Science and Engineering, National Yang Ming Chiao Tung University, Taipei 112, Taiwan; 5 Department of Biomedical Engineering, University of Southern California, Los Angeles, CA 90089-0482, USA; poshunch@usc.edu; 6 Department of Clinical Laboratory, En Chu Kong Hospital, New Taipei City 237, Taiwan; 10036@km.eck.org.tw; 7 Institute of Medicine, Chung Shan Medical University, Taichung 402, Taiwan; hyhsien@csmu.edu.tw; 8 Institute of Clinical Pharmacy and Pharmaceutical Sciences, College of Medicine, National Cheng Kung University, Tainan 701, Taiwan

**Keywords:** antimicrobial susceptibility, longitudinal surveillance, phylogenetic groups, urinary tract infections, virulence factors

## Abstract

*Escherichia coli* causing urinary tract infections (UTIs) are one of the most common outpatient bacterial infections. This study aimed to compare the characteristics of *E. coli* isolated from UTI patients in a single medical center in 2009–2010 (n = 504) and 2020 (n = 340). The antimicrobial susceptibility of *E. coli* was determined by the disk diffusion method. PCRs were conducted to detect phylogenetic groups, ST131, K1 capsule antigen, and 15 virulence factors. Phylogenetic group B2 dominated in our 2009–2010 and 2020 isolates. Moreover, no phylogenetic group E strains were isolated in 2020. *E. coli* isolates in 2020 were more susceptible to amoxicillin, ampicillin/sulbactam, cefuroxime, cefmetazole, ceftazidime, cefoxitin, tetracycline, and sulfamethoxazole/trimethoprim, compared to the isolates in 2009–2010. Extensively drug-resistant (XDR)-*E. coli* in 2009–2010 were detected in groups B1 (5 isolates), B2 (12 isolates), F (8 isolates), and unknown (1 isolate). In 2020, XDR-*E. coli* were only detected in groups A (2 isolates), B2 (5 isolates), D (1 isolate), and F (4 isolates). The prevalence of virulence factor genes *aer* and *fimH* were higher in *E. coli* in 2009–2010 compared to those in 2020. In contrast, *afa* and *sat* showed higher frequencies in *E. coli* isolates in 2020 compared to *E. coli* in 2009–2010.

## 1. Introduction

Urinary tract infection (UTI) is one of the most common outpatient bacterial infections worldwide with a lifetime incidence of 50–60% in adult women and contribute to a substantial financial burden on society [1]. Lower UTI refers to the inflammation and infection of the bladder and urethra [2]. In contrast, upper UTI severely affects the function of kidneys and could be potentially life-threatening when bacteria invade the bloodstream from infected kidneys to cause urosepsis [2]. UTI in young, healthy, non-pregnant, pre-menopausal female patients with anatomically and functionally normal urinary tract is called uncomplicated UTI. However, UTI associated with host risk factors such as host immunodeficiency, urinary tract abnormality, bladder dysfunction in type 2 diabetes, and estrogen deficiency, with increasing colonization and decreasing efficacy of therapy, is defined as complicated UTI [3,4].

The elderly are more susceptible to uropathogenic *Escherichia coli* (UPEC), which is the dominant infectious pathogen in both uncomplicated and complicated UTIs [1,5]. UPEC strains show great diversity in their gene content, virulence factors, genomic organization, and pathogenicity islands [6]. Several virulence factors of UPEC are shown to be related to the pathogenesis of bacterial UTI [1,7,8,9,10]. Wang et al. reported that regarding the bacterial characteristics in diabetic patients with UPEC, the isolated *E. coli* strains had more virulence factor genes, including K1 capsule *neuA*, adhesin *papGII*, afimbrial adhesin *afa*, and hemolysin *hlyA* [11]. Type 1 fimbrial adhesin FimH has a critical role not only in lower UTI pathogenesis but also in kidney infections by acting synergistically with PapGII [8]. Moreover, fimbriae [12], iron acquisition systems [13], iron-regulated gene homologue adhesin Iha [14], ferric aerobactin receptor IutA [15], cytotoxic necrotizing factor 1 (Cnf1) [16], hemolysin (HlyA) [16], uropathogenic specific protein (Usp) [17], and outer membrane protease T (OmpT) [18], are also shown as important virulence factors of *E. coli* in murine uropathogenesis. Carriage of these urovirulence factors is thought to enhance UPEC pathogenicity and is used to measure and categorize clinical UPEC strains isolated from different patient populations [19,20,21]. Moreover, *E. coli* isolated from the elderly have fewer virulence factors compared to those isolated from younger age groups [5].

Antibiotic therapy is an effective approach to reduce the duration of UTI symptoms. First-line antibiotics such as trimethoprim and some β-lactams, or second-line quinolones and amoxicillin in combination with clavulanic acid, are considered effective treatments for UTIs. In addition, carbapenems were considered the last resort treatment for infections caused by extended-spectrum β-lactamases-producing *Enterobacterales*. In the last decade, the extensive use of antibiotics has resulted in the emergence of antibiotic-resistant pathogens and leads to the spread of antibiotic resistance [22]. Importantly, carbapenem-resistant UPEC in community-acquired UTIs was also reported worldwide [23,24].

Although the association of host factors and bacterial virulence genes with the pathogenesis of *E. coli* causing UTIs has been reported [5,25,26], the longitudinal survey to compare the characteristics of *E. coli* isolated from a single medical center to determine the distinct characteristics of UTI-causing *E. coli* at a 10-year interval is rare. In this study, we aimed to compare the bacterial characteristics, including phylogenetic groups, antibiotic susceptibility, and virulence factors, of *E. coli* strains isolated from UTI patients in 2009–2010 and 2020.

## 2. Results

### 2.1. The Different Distribution of Phylogenetic Groups amongst E. coli Isolates at a 10-Year Interval

We previously divided UTI patients into six age groups (≤3, 4–20, 21–40, 41–60, 61–80, and >80 years old) and reported that *E. coli* isolated from the elderly with UTI were more resistant to antimicrobial agents and had fewer virulence factors compared to *E. coli* isolated from the younger age groups [5]. To exclude the patient’s age effect, we randomly selected 504 and 340 *E. coli* isolated from six patient age groups with UTI in 2009–2010 and 2020, respectively (Table 1). The average age of patients with *E. coli* causing UTI in 2009–2010 was 44.47 years, with 73.4% of these *E. coli* were isolated from women (Table 1). The average age of patients with *E. coli* causing UTI in 2020 was 47.6 years, and 65.3% of these *E. coli* were isolated from women (Table 1).

Phylogenetic analysis has classified *E. coli* strains into eight groups (A, B1, B2, C, D, E, F, and clade I) [27]. We next examined the distribution of phylogenetic groups among our 844 isolates. The results showed that the distribution of phylogenetic groups in *E. coli* was different in 2009–2010 and 2020 (*p* < 0.001). Phylogenetic group B2 dominated in our 2009–2010 isolates (313 isolates, 62.1%), followed by group B1 (44 isolates, 8.7%), group D (43 isolates, 8.5%), group C (33 isolates, 6.5%), group F (32 isolates, 6.3%), group E (15 isolates, 3.0%), unknown (14 isolates, 2.8%), group A (9 isolates, 1.8%), and clade I (1 isolate, 0.2%). Phylogenetic group B2 was also dominated in our 2020 isolates (234 isolates, 68.8%), followed by group D (29 isolates, 8.5%), group B1 (22 isolates, 6.5%), group F (19 isolates, 5.6%), group A (17 isolates, 5.0%), unknown (10 isolates, 2.9%), group C (6 isolates, 1.8%), and clade I (3 isolates, 0.9%). Interestingly, no phylogenetic group E was isolated in 2020. In summary, the predominant phylogenetic group was B2 (547/844, 64.8%), followed by D (72/844, 8.5%), B1 (66/844, 7.8%), F (51/844, 6.0%), C (38/844, 4.6%), A (26/844, 3.1%), unknown (24/844, 2.8%), and clade I (4/844 0.5%). *E. coli* sequence type 131 (ST131) has emerged rapidly to become the most drug-resistant and prevalent extraintestinal pathogenic *E. coli* clone in circulation worldwide. Therefore, we next determined the prevalence of ST131 in our 2009–2010 and 2020 isolates by PCR with ST131 specific primers. The results showed that 69 (13.7%) and 33 (9.7%) of 2009–2010 and 2020 *E. coli* were ST131, respectively (*p* = 0.082).

### 2.2. Antimicrobial Susceptibility of E. coli Isolated from UTI Patients

We further compared the antimicrobial susceptibility (21 antimicrobials divided into 11 categories) of *E. coli* isolates collected in 2009–2010 and 2020. We found all UTI isolates were susceptible to tigecycline. Interestingly, *E. coli* isolates in 2020 were more susceptible to amoxicillin (*p* = 0.011), ampicillin/sulbactam (*p* = 0.001), cefuroxime (*p* = 0.018), cefmetazole (*p* < 0.001), ceftazidime (*p* < 0.001), cefoxitin (*p* < 0.001), tetracycline (*p* = 0.011), and sulfamethoxazole/trimethoprim (*p* = 0.010), compared to the isolates in 2009–2010 (Table 2). In general, our UTI isolates were highly susceptible (> 90% susceptibility) to amikacin, piperacillin/tazobactam, imipenem, ertapenem, meropenem, but were resistant (< 60% susceptibility) to ampicillin, ciprofloxacin, tetracycline, and sulfamethoxazole/trimethoprim (Table 2).

We next determined whether the antimicrobial susceptibility of *E. coli* isolates was associated with phylogenetic groups (Table 3). In this study, we determined the antimicrobial susceptibility of *E. coli* isolates to 21 antimicrobials (divided into 11 categories), and then we classified our isolates into multidrug-resistant (MDR, non-susceptible to ≥ one agent in ≥ three antimicrobial categories), extensively drug-resistant (XDR, non-susceptible to ≥ one agent in all but ≤ two categories), and pandrug-resistant (PDR, non-susceptible to all antimicrobial agents), according to the previous study [28]. We found 279 MDR-*E. coli* (279/504, 55.4%) and 26 XDR-*E. coli* (26/504, 5.2%) in the 2009–2010 collection, and 187 MDR-*E. coli* (187/340, 55.0%) and 12 XDR-*E. coli* (12/340, 3.5%) in the 2020 collection (Table 3). No PDR-*E. coli* was identified in our 844 isolates. Moreover, there was no significant difference in the prevalence of MDR- and XDR-*E. coli* in 2009–2010 compared to 2020 (*p* = 0.496).

We further determined the distribution of MDR- (187/340, 55.0%) and XDR-*E. coli* among different phylogenetic groups in 2009–2010 and 2020 (Table 3). The results showed a significant difference in the distribution of MDR- and XDR-*E. coli* with phylogenetic groups between the 2009–2010 and 2020 collections (*p* < 0.001). In 2009–2010, 26 XDR-*E. coli* were identified in phylogenetic groups B1 (five isolates), B2 (12 isolates), F (eight isolates), and unknown (one isolate). In contrast, 12 XDR-*E. coli* were only identified in phylogenetic groups A (two isolates), B2 (five isolates), D (one isolate), and F (four isolates), in 2020 (Table 3). In addition, 87.9%, 80.0%, and 79.1%, of phylogenetic groups C, E, and D isolates, were MDR-*E. coli*, in 2009–2010 (Table 3). In contrast, 100%, 83.3%, and 80.0%, of phylogenetic groups clade I, C, and F isolates, were MDR-*E. coli*, in 2020 (Table 3).

### 2.3. Antimicrobial Susceptibility of E. coli Isolated from UTI Patients Is Associated with Patient Age

We previously reported that *E. coli* strains isolated from patient age group >80 were more resistant to amoxicillin in combined with clavulanate, cefazolin, cefixime, cefmetazole, ceftriaxone, cefuroxime, ciprofloxacin, levofloxacin, than the average resistant rate [5]. In contrast, *E. coli* strains isolated from patient age group ≤3 were more susceptible to amoxicillin in combined with clavulanate, cefixime, cefuroxime, ciprofloxacin, levofloxacin, and piperacillin/tazobactam, than the average resistant rate [5]. The distribution of antimicrobial resistance of *E. coli* isolated from different host age groups was shown in Table S1. Overall, *E. coli* isolated from the elderly were more resistant to cefazolin, cefuroxime, ceftriaxone, ciprofloxacin, and levofloxacin, in 2009–2010 and 2020 (*p* < 0.05) (Table S1). We next determined the antimicrobial susceptibility of *E. coli* isolated from two extreme age groups of UTI patients in 2009–2010 and 2020 (Table 4). 

In 2009–2010 and 2020, *E. coli* strains isolated from patient age group >80 were more resistant to cefazolin, cefuroxime, ceftriaxone, ciprofloxacin, and levofloxacin (*p* < 0.05), than the *E. coli* isolated from the patient age group ≤3 (Table 4). In 2009–2010, *E. coli* strains isolated from patient age group >80 were more resistant to amoxicillin (*p* = 0.007), ampicillin/sulbactam (*p* = 0.001), cefmetazole (*p* < 0.001), and ceftazidime (*p* = 0.006) (Table 4). Interestingly, *E. coli* strains isolated from patient age group ≤3 were more resistant to sulfamethoxazole/trimethoprim (53.3% vs 35.8%, *p* = 0.047), than the *E. coli* strains isolated from age group >80, in 2020 (Table 4). The distribution of MDR- and XDR-*E. coli* among these two patient age groups was not significantly different, in both 2009–2010 and 2020. Moreover, we found isolates in age group >80 in 2020 were more susceptible to ampicillin/sulbactam (*p* = 0.048), cefmetazole (*p* = 0.001), cefoxitin (*p* = 0.004), and sulfamethoxazole/trimethoprim (*p* = 0.002), compared to isolates in age group >80 in 2009–2010 (Table 4).

### 2.4. The Different Prevalence of Bacterial Virulence Factors in E. coli Isolated from UTI Patients at a 10-Year Interval

To characterize the *E. coli* isolated from UTI patients in 2009–2010 and 2020, we determined the presence of K1 capsule antigen and 15 virulence factors in 844 isolates by PCR (Table 5). Overall, virulence factor genes *aer*, *usp*, *ompT*, and *fimH*, were found in more than 60% of 844 isolates (Table 5). The results showed that the prevalence of virulence factor genes *aer* (64.3% vs 55.6, *p* = 0.011) and *fimH* (97.2% vs 89.1%, *p* < 0.001), were higher in *E. coli* in 2009–2010, compared to those in 2020 (Table 5). In contrast, *afa* (63.8% vs 56.3%, *p* = 0.030) and *sat* (40.9% vs 32.7%, *p* = 0.016), showed higher frequencies in *E. coli* in 2020, compared to *E. coli* in 2009–2010 (Table 5).

The distribution of virulence facctors in *E. coli* isolated from different host age groups was shown in Table S2. The prevalence of *papGII*, *cnfI*, *usp*, and *ompT*, were decreased in *E. coli* isolated from the elderly in both 2009–2010 and 2020 (*p* < 0.05) (Table S2). When the strains isolated from patient age groups ≤3 and >80 were compared, we found strains isolated from the elderly age group had fewer virulence factors, including *papGII* (*p* = 0.001), *papGIII* (*p* = 0.028), *cnf1* (*p* = 0.015), *usp* (*p* = 0.009), and *hlyA* (*p* = 0.001), compared to the *E. coli* isolated from patient age group ≤3, in 2009–2010 (Table 6). In 2020, the prevalence of virulence factors such as *papGII* (*p* < 0.001), *foc* (*p* < 0.038), *cnf1* (*p* = 0.005), and *aer* (*p* = 0.024), were lower in the patient age group >80 compared to ≤3 age group (Table 6). Moreover, we found isolates in age group ≤3 in 2020 have less *papGIII* (6.7% vs 22.7%, *p* = 0.011) but more *afa* (75.0% vs 52.0%, *p* = 0.006), compared to isolates in age group ≤3 in 2009–2010 (Table 6).

## 3. Discussion

The longitudinal survey to investigate the distinct characteristics of *E. coli* isolated from a single medical center at a 10-year interval is rare. In this study, we compared the bacterial characteristics, including phylogenetic groups, antimicrobial susceptibility, and virulence factors, of *E. coli* strains isolated from patients with UTI in 2009–2010 and 2020. We applied modified phylogenetic analysis to classify *E. coli* strains into eight groups in this study. Although phylogenetic group B2 was predominant in our 2009–2010 (62.1%) and 2020 (68.8%) *E. coli* isolates, we found 38 (4.6%) and 51 (6.0%) isolates in our 844 isolates belonged to phylogenetic groups C and E, respectively. Moreover, the results showed a decrease of groups C and E at a 10-year interval. Iranpour et al. reported the predominant phylogenetic group was B2 (39.3%), followed by unknown (27.1%), E (9.3%), C and clade I (each 6.4%), B1 (5%), F and D (each 2.9%), and A (0.7%) in *E. coli* causing UTI in Iran [29]. These results suggest the geographical difference of characteristics of *E. coli* isolated from UTI patients in different countries. Our previous study showed that phylogenetic group B2 dominated in UTI isolates (541/907, 59.6%), followed by group D (188/907, 20.7%), group A (95/907, 10.5%), and group B1 (83/907, 10.5), according to old phylogenetic analysis method [30]. Compared to our previous report [30], we found a significant decrease of group D (20.7% vs. 8.5%) and group A (10.5% and 3.1%) isolates in this study. Gordon et al. reported that 21% of *E. coli* strains were either incorrectly assigned to a phylogenetic group by the old triplex method or could not be assigned to one of the phylogenetic groups A, B1, B2, D, or E using the MLST data [31]. Therefore, this inconsistency may result from the incorrect assignment of *E. coli* isolates to a phylogenetic group, and thus the application of modified phylogenetic analysis to precisely classify *E. coli* strains is necessary.

Banerjee et al. reported ST131 was a dominant, antimicrobial-resistant clonal group associated with healthcare settings, elderly hosts, and persistent or recurrent symptoms [32]. Our findings show a stable existence of ST131 in a medical center in Taiwan. However, the prevalence of ST131 in patients with recurrent UTI was 17.2% (unpublished data), higher than the prevalence of this clone in UTI patients’ first episode in this study. We found 29 (87.9%) and 5 (83.3%) of phylogenetic group C isolates in 2009–2010 and 2020, respectively, were MDR-*E. coli* (Table 3). In contrast, only 140 (44.7%) and 118 (50.44%) B2 isolates in 2009–2010 and 2020, respectively, were MDR-*E. coli* (Table 3). 

Recently, Chakraborty et al. collected 33 *bla*_NDM-5_-producing *E. coli* isolates which were highly resistant to β-lactams, including novel β-lactam/β-lactamase inhibitor combinations (ceftazidime/avibactam, imipenem/relebactam, and meropenem/vaborbactam). They found these isolates were assigned to different sequence types (STs) and indicated a predominance of isolates exhibiting ST167 in Switzerland and Germany (*n* = 10) (phylogenetic group C), followed by ST405 (group E), ST1284 (group C), and ST361 (group C) [33]. These results suggest that the new assigned phylogenetic groups, C and E, may be responsible for the spread of MDR-*E. coli* worldwide. Therefore, the characteristics of *E. coli* phylogenetic groups C and E are worth investigating and comparing with other groups.

We found *E. coli* isolated in 2020 were, in general, more susceptible to many antimicrobials. These results suggest the decrease of antimicrobial resistance in 2020 may result from the implementation of the national antimicrobial stewardship program from 2013 in Taiwan. In addition, we showed that *E. coli* strains isolated from age group >80 were more resistant to most commonly used antimicrobial agents than strains isolated from age group ≤3 (Table 4). These results are consistent with our previous findings [5]. In addition, Pulcini et al. also revealed that the elderly people in nursing homes had a risk around 40% higher than their community-dwelling peers of having antibiotic-resistant *Enterobacteriaceae* cultured from their urine samples [34]. Moreover, *E. coli* showed resistance to amoxicillin/clavulanate, nalidixic acid, ofloxacin, ciprofloxacin, ceftriaxone, and ESBLs were all more prevalent in nursing home samples than in community samples [34]. This observation may be caused by the high frequency and long-term use of antimicrobials in the elderly compared to the younger generation. As a result, antimicrobial stewardship and infection prevention and control programs should be tightly implemented in the elderly.

Consistent with previous reports [35,36], we found the high frequency of type 1 fimbrial adhesin gene *fimH* (94.0%) in our 844 isolates (Table 5). These results suggest a critical role of FimH in establishing successful colonization of *E. coli* in the urinary tract. Our results showed a change in the prevalence of virulence factors in *E. coli* isolates in 2020 compared to 2009–2010 (Table 5). The significance of this evolutionary tract in *E. coli* uropathogenesis remains to be studied. In addition, the effect of different combinations of virulence factors in urinary tract colonization of *E. coli* is worth investigating. Here, our results revealed a decrease of virulence factors in *E. coli* isolated from the elderly compared to the younger generation (Table 6), which were consistent with our previous report, which showed the prevalence of virulence factors and antibiotic resistance of *E. coli* causing UTI were associated with patient age [5]. *E. coli* isolated from the elderly were more resistant to antimicrobials and had fewer virulence factors [5]. The role(s) of specific virulence factors in *E. coli* causing UTI in different patient age groups remains to be studied experimentally. Moreover, direct evidence demonstrating that the elderly are more vulnerable to low virulent *E. coli* caused by the decline of host immune protection or increase in host risk factors is still lacking.

In this study, we compared the characteristics of *E. coli* isolated from UTI patients in a single medical center at a 10-year interval to determine the regional evolutionary change of *E. coli*. We found a decrease in antimicrobial resistance and a difference in phylogenetic group composition and virulence factor distribution of *E. coli* at a 10-year interval. However, the driving force behind these phenotypic and genotypic changes among *E. coli* causing UTI at a 10-year interval remains to be investigated. In addition, whether these changes are associated with the virulence of *E. coli* is unclear. Moreover, our results showed that the characteristics of *E. coli* isolated from UTI patients were strongly associated with patient age. *E. coli* isolated from the elderly were more resistant to antimicrobials and had fewer virulence factors. Accordingly, the distinct characteristics of *E. coli* isolated from different age groups revealed in this study may be beneficial for clinical physicians to precisely control *E. coli*-caused UTI in different age groups of patients in the future.

## 4. Materials and Methods

### 4.1. Sampling and Isolation of E. coli

*E. coli* isolates were recovered from patients with UTIs at National Cheng Kung University hospital during 2009–2010 and 2020. This study was approved by the NCKUH Research Ethics Committee (B-ER-110-144). *E. coli* isolates were identified in the clinical laboratory by colony morphology, Gram stain, biochemical tests, and the Vitek system (bioMérieux, Marcy l’Etoile, France) according to the manufacturer’s recommendations. A total of 504 and 340 non-duplicate *E. coli* isolates were collected in 2009–2010 and 2020, respectively. *E. coli* isolates were stored at −80 °C in lysogeny broth (LB) containing 20% glycerol (*v*/*v*) until tested.

### 4.2. Virulence Factors Identification

K1 capsule antigen gene and 15 virulence factor genes of *E. coli* were detected by PCR according to previous studies [5,25,37]. Primer pairs specific for the K1 capsule gene (*neuA*), 3 PapG adhesion genes of P-fimbriae (*papG* class I to III), type 1 fimbrial adhesins (*fimH*), S-/F1C-fimbriae (*sfa*/*foc*), afimbrial adhesins (*afa*), iron-regulated gene homologue adhesin (*iha*), hemolysin (*hlyA*), cytotoxic necrotizing factor 1 (*cnf1*), catecholate siderophore receptor (*iroN*), ferric aerobactin receptor (*iutA*), outer membrane protease T (*ompT*), and uropathogenic specific protein (*usp*), are listed in Table S3. Positive and negative control strains for the traits of interest were included in each assay.

### 4.3. Phylogenetic Grouping and Escherichia coli Sequence Type 131 (ST131) Detection

Based on PCR amplification patterns of specific genetic markers (*arpA*, *chuA*, *trpA*, *yjaA*, and TSPE4.C2), *E. coli* strains were divided into eight phylogenetic groups: A, B1, B2, C, D, E, F, and clade I, according to the previous study [27]. The primers used for phylogenetic typing are listed in Table S3.

Primers ST131_for (5’-GACTGCATTTCGTCGCCATA-3’) and ST131_rev (5’-CCGGCGGCATCATAATGAAA-3’) in combined purified genomic DNA as a template were used to perform PCR to detect *E. coli* ST131, according to the previous study [38]. Amplification mixtures were performed with the following cycling conditions: an initial denaturation at 94 °C for 3 min, 30 cycles of 94 °C for 30 s, 60 °C for 30 s, and 72 °C for 30 s, and one final cycle of 72 °C for 5 min.

### 4.4. Antimicrobial Susceptibility Testing

The antimicrobial susceptibility was experimentally determined through disk diffusion assay against 21 antibiotics, including amikacin (30 μg), amoxicillin (30 μg), ampicillin (10 μg), ampicillin/sulbactam (10 μg/10 μg), cefazolin (30 μg), cefepime (30 μg), cefmetazole (30 μg), cefoxitin (30 μg), ceftazidime (30 μg), ceftriaxone (30 μg), cefuroxime (30 μg), ciprofloxacin (5 μg), ertapenem (10 μg), gentamicin (10 μg), imipenem (10 μg), meropenem (10 μg), levofloxacin (5 μg), piperacillin/tazobactam (100 μg/10 μg), sulfamethoxazole/trimethoprim (23.27 μg/1.25 μg), tetracycline (30 μg), and tigecycline (15 μg) (BD BBL Sensi-Disc, Becton, Dickinson and Company, Sparks, MD, USA) following the Clinical and Laboratory Standards Institute (CLSI) guidelines [39]. *E. coli* ATCC 25922 was used as our quality control strain. The interpretation of resistance to these antimicrobial agents was determined according to the recommendations of the CLSI [39].

### 4.5. Statistical Analysis 

Pearson’s Chi-square tests or Student *t*-tests were used for comparing categorical variables. All statistical analyses were performed using IBM SPSS statistics version 24.0 (IBM Corporation, Armonk, NY, USA). A *p*-value < 0.05 was taken as significant.

## Figures and Tables

**Table 1:** The distribution of the gender and average age of UTI patients in 2009–2010 and 2020.

	Year of Isolation	
	2009–2010 (n = 504)	2020 (n = 340)
Average age (year)	44.47	47.76
Age group (years old), n (%)		
≤3	75 (14.9)	60 (17.6)
4–20	62 (12.3)	17 (5.0)
21–40	97 (19.2)	62 (18.2)
41–60	89 (17.7)	66 (19.4)
61–80	92 (18.3)	68 (20.0)
>80	89 (17.7)	67 (19.7)
Gender, n (%)		
Female	370 (73.4)	222 (65.3)
Male	134 (26.6)	118 (34.7)

**Table 2:** Antimicrobial susceptibility of UTI *E. coli* isolates in 2009–2010 and 2020.

	Year of Isolation						
	2009–2010 (n = 504)			2020 (n = 340)			*p*-Value
Antimicrobial Category and Agents							
Aminoglycoside	S	I	R	S	I	R	
AN	486 (98.4)	2 (0.4)	6 (1.2)	337 (99.1)	1 (0.3)	2 (0.6)	0.376
GM	360 (71.4)	14 (2.8)	130 (25.8)	263 (77.4)	7 (2.1)	70 (20.6)	0.055
Penicillins							
AM	115 (22.8)	1 (0.2)	388 (77.0)	89 (26.2)	6 (1.8)	245 (72.1)	0.264
AMC	329 (65.3)	44 (8.7)	131 (26)	250 (73.5)	44 (12.9)	46 (13.5)	0.011
Penicillins + β-lactamase inhibitors							
SAM	335 (66.5)	62 (12.3)	107 (21.2)	265 (77.9)	24 (7.1)	51 (15.0)	<0.001
TZP	483 (95.8)	16 (3.2)	5 (1.0)	331 (97.4)	6 (1.8)	3 (0.9)	0.242
Carbapenems							
IPM	501 (99.4)	3 (0.6)	0 (0)	336 (98.8)	2 (0.6)	2 (0.6)	0.361
ETP	497 (98.6)	3 (0.6)	4 (0.8)	338 (99.4)	1 (0.3)	1 (0.3)	0.267
MEM	502 (99.6)	0 (0)	2 (0.4)	338 (99.4)	0 (0)	2 (0.6)	0.691
Non-extended spectrum cephalosporins							
CZ	338 (67.1)	0 (0)	166 (32.9)	248 (72.9)	0 (0)	92 (27.1)	0.069
CXM	340 (67.5)	35 (6.9)	129 (25.6)	255 (75.0)	7 (2.1)	78 (22.9)	0.018
CMZ	422 (83.7)	29 (5.8)	53 (10.5)	323 (95.0)	1 (0.3)	16 (4.7)	<0.001
Extended-spectrum cephalosporins							
CRO	343 (68.1)	18 (3.6)	143 (28.4)	252 (74.1)	4 (1.2)	84 (24.7)	0.058
CAZ	364 (72.2)	31 (6.2)	109 (21.6)	281 (82.6)	25 (7.4)	34 (10.0)	<0.001
FEP	432 (85.7)	29 (5.8)	43 (8.5)	289 (85.0)	16 (4.7)	35 (10.3)	0.773
Cephamycins							
FOX	380 (75.4)	19 (3.8)	105 (20.8)	310 (91.2)	6 (1.8)	24 (7.1)	<0.001
Fluoroquinolones							
CIP	293 (58.1)	36 (7.1)	175 (34.7)	192 (56.5)	27 (7.9)	121 (35.6)	0.631
LVX	335 (66.5)	8 (1.6)	161 (31.9)	223 (65.6)	5 (1.5)	112 (32.9)	0.791
Tetracyclines							
TE	210 (41.7)	23 (4.6)	271 (53.8)	172 (50.6)	10 (2.9)	158 (46.5)	0.011
Glycylcyclines							
TIG	504 (100)	0 (0)	0 (0)	340 (100)	0 (0)	0 (0)	-
Folate pathway inhibitors							
SXT	242 (48.0)	1 (0.2)	261 (51.8)	194 (57.1)	0 (0)	146 (42.9)	0.010

Abbreviations: AM, ampicillin; AMC, amoxicillin; AN, amikacin; CAZ ceftazidime, CIP, ciprofloxacin; CMZ, cefmetazole; CRO, ceftriaxone; CXM, cefuroxime; CZ, cefazolin; ETP, ertapenem; FEP, cefepime; FOX, cefoxitin; GM, gentamicin; LVX, levofloxacin; IPM, imipenem; MEM meropenem; SAM, ampicillin/sulbactam; SXT, sulfamethoxazole/trimethoprim; TE, tetracycline; TIG, tigecycline; TZP, piperacillin/tazobactam; S, susceptible; I, intermediate resistant; R, resistant.

**Table 3:** The distribution of multidrug-resistant and extensively drug-resistant *E. coli* in 2009–2010 and 2020.

	Year of Isolation					
	2009–2010 (n = 504)			2020 (n = 340)		
Drug-Resistant Isolate, n (%)	Non-MDR or XDR	MDR	XDR	Non-MDR or XDR	MDR	XDR
A	3 (33.3)	6 (66.7)	0 (0)	3 (17.6)	12 (70.6)	2 (11.8)
B1	7 (15.9)	32 (72.7)	5 (11.4)	12 (54.5)	10 (45.5)	0 (0)
B2	161 (51.4)	140 (44.7)	12 (3.9)	111 (47.4)	118 (50.4)	5 (2.2)
C	4 (12.1)	29 (87.9)	0 (0)	1 (16.7)	5 (83.3)	0 (0)
D	9 (20.9)	34 (79.1)	0 (0)	11 (37.9)	17 (58.6)	1 (3.5)
E	3 (20.0)	12 (80.0)	0 (0)	0 (0)	0 (0)	0 (0)
F	6 (18.8)	18 (56.3)	8 (24.9)	1 (5.3)	14 (73.7)	4 (21.0)
Clade I	1 (100)	0 (0)	0 (0)	0 (0)	3 (100)	0 (0)
Unknown	5 (35.7)	8 (57.1)	1 (7.2)	2 (20.0)	8 (80.0)	0 (0)
Total	199 (39.4)	279 (55.4)	26 (5.2)	141 (41.5)	187 (55.0)	12 (3.5)

Abbreviations: MDR, multidrug-resistant; XDR, extensively drug-resistant.

**Table 4:** The distribution of antimicrobial-resistant *E. coli* in the age groups ≤3 and >80 in 2009–2010 and 2020.

	Age Group (Years Old) in 2009–2010			Age Group (Years Old) in 2020			*p*-Value	
Antimicrobial Category and Agents	≤3 (n = 75)	>80 (n = 89)	*p*-Value	≤3 (n = 60)	>80 (n = 67)	*p*-Value	≤3 Age Group ^a^	>80 Age Group ^b^
	I + R	I + R		I + R	I + R			
Aminoglycoside								
AN	1 (1.3)	2 (2.2)	0.664	0 (0)	1 (1.5)	0.342	0.369	0.734
GM	25 (33.3)	25 (28.1)	0.467	17 (28.3)	14 (20.9)	0.330	0.533	0.304
Penicillins								
AM	62 (82.7)	74 (83.1)	0.935	44 (73.3)	51 (76.1)	0.718	0.189	0.276
AMC	17 (22.7)	38 (42.7)	0.007	16 (26.7)	22 (32.8)	0.448	0.591	0.210
Penicillins + β-lactamase inhibitors								
SAM	14 (18.7)	39 (43.8)	0.001	12 (20.0)	19 (28.4)	0.274	0.845	0.048
TZP	1 (1.3)	6 (6.7)	0.088	0 (0)	2 (3.0)	0.177	0.369	0.292
Carbapenems								
IPM	0 (0)	0 (0)	-	0 (0)	2 (3.0)	0.177	-	0.101
ETP	1 (1.3)	2 (2.2)	0.664	0 (0)	0 (0)	-	0.369	0.217
MEM	1 (1.3)	1 (1.1)	0.903	0 (0)	1 (1.5)	0.342	0.369	0.839
Non-extended spectrum cephalosporins								
CZ	15 (20.0)	39 (43.8)	0.001	15 (25.0)	31 (46.3)	0.013	0.487	0.761
CXM	15 (20.0)	37 (41.6)	0.003	14 (23.3)	28 (41.8)	0.027	0.639	0.978
CMZ	2 (2.7)	22 (24.7)	<0.001	1 (1.7)	6 (9.0)	0.072	0.695	0.011
Extended-spectrum cephalosporins								
CRO	16 (21.3)	37 (41.6)	0.006	14 (23.3)	31 (46.3)	0.007	0.781	0.558
CAZ	12 (16.0)	31 (34.8)	0.006	10 (16.7)	16 (23.9)	0.314	0.917	0.140
FEP	9 (12.0)	19 (21.3)	0.113	10 (16.7)	15 (22.4)	0.418	0.438	0.876
Cephamycins								
FOX	8 (10.7)	30 (33.7)	<0.001	3 (4.9)	9 (13.4)	0.105	0.232	0.004
Fluoroquinolones								
CIP	20 (26.7)	51 (57.3)	<0.001	20 (33.3)	39 (58.2)	0.005	0.399	0.910
LVX	15 (20.0)	43 (48.3)	<0.001	16 (26.7)	36 (53.7)	0.002	0.360	0.503
Tetracyclines								
TE	46 (61.3)	52 (58.4)	0.705	29 (48.3)	33 (49.3)	0.917	0.131	0.255
Glycylcyclines								
TIG	0 (0)	0 (0)	-	0 (0)	0 (0)	-	-	-
Folate pathway inhibitors								
SXT	41 (54.7)	54 (60.7)	0.438	32 (53.3)	24 (35.8)	0.047	0.877	0.002
Drug-resistance			0.052			0.145	0.467	0.855
MDR	39 (52.0)	57 (64.0)		36 (60.0)	40 (59.7)			
XDR	1 (1.3)	5 (5.6)		0 (0)	4 (6.0)			

^a^ Statistical results for the distribution of virulence factors in patient age group ≤3 years old in 2009–2010 compared to 2020. ^b^ Statistical results for the distribution of virulence factors in patient age group >80 years old in 2009–2010 compared to 2020. Abbreviations: AM, ampicillin; AMC, amoxicillin; AN, amikacin; CAZ ceftazidime, CIP, ciprofloxacin; CMZ, cefmetazole; CRO, ceftriaxone; CXM, cefuroxime; CZ, cefazolin; ETP, ertapenem; FEP, cefepime; FOX, cefoxitin; GM, gentamicin; LVX, levofloxacin; IPM, imipenem; MEM meropenem; SAM, ampicillin/sulbactam; SXT, sulfamethoxazole/trimethoprim; TE, tetracycline; TIG, tigecycline; TZP, piperacillin/tazobactam; I, intermediate resistant; R, resistant; MDR, multidrug-resistant; XDR, extensively drug-resistant.

**Table 5:** The distribution of virulence factor genes in UTI *E. coli* in 2009–2010 and 2020.

Virulence Factor Genes	2009–2010 (n = 504)	2020 (n = 340)	Total (n = 844)	*p*-Value
*papGI*	0 (0)	1 (0.3)	1 (0.1)	0.223
*papGII*	144 (28.6)	77 (22.6)	221 (26.2)	0.055
*papGIII*	85 (16.9)	41 (12.1)	126 (14.9)	0.055
*sfa*	43 (8.5)	43 (12.6)	86 (10.2)	0.053
*foc*	53 (10.5)	40 (11.8)	93 (11.0)	0.570
*cnf1*	112 (22.2)	92 (27.1)	204 (24.2)	0.107
*aer*	324 (64.3)	189 (55.6)	513 (60.8)	0.011
*usp*	318 (63.1)	200 (58.8)	518 (61.4)	0.211
*iha*	180 (35.7)	111 (32.6)	291 (34.5)	0.358
*ompT*	400 (79.4)	253 (74.4)	653 (77.4)	0.092
*afa*	284 (56.3)	217 (63.8)	501 (59.4)	0.030
*iRONE*	20 (40.3)	121 (35.6)	324 (38.4)	0.169
*fimH*	490 (97.2)	303 (89.1)	793 (94.0)	<0.001
*hly*	125 (24.8)	105 (30.9)	230 (27.3)	0.052
*sat*	165 (32.7)	139 (40.9)	304 (36.0)	0.016
*K1*	126 (25.0)	75 (22.1)	201 (23.8)	0.325

**Table 6:** The distribution of virulence factors in *E. coli* in ≤3 and >80 age groups in 2009–2010 and 2020.

	Age Group (Years Old) in 2009–2010			Age Group (Years Old) in 2020			*p*-Value	
Virulence Factor Genes ^a^	≤3 (n = 75)	>80 (n = 89)	*p*-Value	≤3 (n = 60)	>80 (n = 67)	*p*-Value	≤3 Age Group ^a^	>80 Age Group ^b^
*papGI*	0 (0)	0 (0)	-	0 (0)	0 (0)	-	-	-
*papGII*	35 (46.7)	19 (21.3)	0.001	30 (50.0)	10 (14.9)	<0.001	0.700	0.307
*papGIII*	17 (22.7)	9 (10.1)	0.028	4 (6.7)	8 (11.9)	0.310	0.011	0.717
*sfa*	10 (13.3)	9 (10.1)	0.521	9 (15.0)	8 (11.9)	0.613	0.782	0.717
*foc*	8 (10.7)	6 (6.7)	0.370	12 (20.0)	5 (7.5)	0.038	0.129	0.862
*cnf1*	26 (34.7)	16 (18.0)	0.015	23 (38.3)	11 (16.4)	0.005	0.660	0.799
*aer*	56 (74.7)	62 (69.7)	0.477	47 (78.3)	40 (59.7)	0.024	0.619	0.195
*usp*	56 (74.7)	49 (55.1)	0.009	43 (71.7)	39 (58.2)	0.113	0.695	0.694
*iha*	37 (49.3)	36 (40.4)	0.254	24 (40.0)	27 (40.3)	0.973	0.279	0.985
*ompT*	65 (86.7)	68 (76.4)	0.094	50 (83.3)	54 (80.6)	0.689	0.588	0.530
*afa*	39 (52.0)	50 (56.2)	0.592	45 (75.0)	40 (59.7)	0.067	0.006	0.659
*iRONE*	31 (41.3)	29 (32.6)	0.247	24 (40.0)	19 (28.4)	0.166	0.876	0.571
*fimH*	74 (98.7)	84 (94.4)	0.145	56 (93.3)	61 (91.0)	0.633	0.103	0.420
*hly*	33 (44.0)	17 (19.1)	0.001	25 (41.7)	18 (26.9)	0.078	0.786	0.250
*sat*	36 (48.0)	34 (38.2)	0.206	28 (46.7)	29 (43.3)	0.702	0.877	0.522
*K1*	18 (24.0)	17 (19.1)	0.446	11 (18.3)	17 (25.4)	0.339	0.426	0.348

^a^ Statistical results for the distribution of virulence factors in patient age group ≤ 3 years old in 2009–2010 compared to 2020. ^b^ Statistical results for the distribution of virulence factors in patient age group >80 years old in 2009–2010 compared to 2020.

## Supplementary Materials

The following are available online at https://www.mdpi.com/article/10.3390/pathogens10091156/s1, Table S1: The distribution of antimicrobial-resistant *E. coli* in different age groups in 2009–2010 and 2020, Table S2: The distribution of virulence factors in *E. coli* in different age groups in 2009–2010 and 2020, Table S3: Oligonucleotide primers used in this study.
