# Supplementary material for: Distinct Characteristics of Escherichia coli Isolated from Patients with Urinary Tract Infections in a Medical Center at a Ten-Year Interval
Source: Pathogens. 2021 Sep 8;10(9):1156. doi: 10.3390/pathogens10091156 (PMC8469484; doi:10.3390/pathogens10091156)
Supplement: Supplementary file 1 [file pathogens-10-01156-s001.zip › Table S1-S3 20210924.pdf]

Table S1. The distribution of antimicrobial-resistant *E. coli* in different age groups in 2009-2010 and 2020.

Antimicrobial category and agents	Age group (years old) in 2009-2010						Age group (years old) in 2020					
	≤3 (n=75)	4-20 (n=62)	21-40 (n=97)	41-60 (n=89)	61-80 (n=92)	>80 (n=89)	≤3 (n=60)	4-20 (n=17)	21-40 (n=62)	41-60 (n=66)	61-80 (n=68)	>80 (n=67)
	I+R	I+R	I+R	I+R	I+R	I+R	I+R	I+R	I+R	I+R	I+R	I+R
Aminoglycoside												
AN	1 (1.3)	0 (0)	0 (0)	1 (1.1)	4 (4.3)	2 (2.2)	0 (0)	0 (0)	0 (0)	1 (1.5)	1 (1.5)	1 (1.5)
GM[a]	25 (33.3)	13 (21.0)	12 (12.4)	29 (32.6)	40 (43.5)	25 (28.1)	17 (28.3)	4 (23.5)	12 (19.4)	11 (16.7)	19 (27.9)	14 (20.9)
Penicillins												
AM[a]	62 (82.7)	46 (74.2)	59 (60.8)	66 (74.2)	82 (89.1)	74 (83.1)	44 (73.3)	10 (58.8)	43 (69.3)	50 (75.8)	53 (77.9)	51 (76.1)
AMC[a]	17 (22.7)	12 (19.4)	16 (16.5)	38 (42.7)	54 (58.7)	38 (42.7)	16 (26.7)	3 (17.6)	14 (22.6)	14 (21.2)	21 (30.9)	22 (32.8)
Penicillins + β-lactamase inhibitors												
SAM[a]	14 (18.7)	13 (21.0)	19 (19.6)	36 (40.4)	48 (52.2)	39 (43.8)	12 (20.0)	4 (23.5)	12 (19.4)	12 (18.2)	16 (23.5)	19 (28.4)
TZP[a]	1 (1.3)	0 (0)	1 (1.0)	5 (5.6)	8 (8.7)	6 (6.7)	0 (0)	0 (0)	1 (1.6)	1 (1.5)	5 (7.4)	2 (3.0)
Carbapenems												
IPM	0 (0)	0 (0)	0 (0)	1 (1.1)	2 (2.2)	0 (0)	0 (0)	0 (0)	0 (0)	1 (1.5)	1 (1.5)	2 (3.0)
ETP	1 (1.3)	0 (0)	0 (0)	2 (2.2)	2 (2.2)	2 (2.2)	0 (0)	0 (0)	0 (0)	2 (3.0)	0 (0)	0 (0)
MEM	1 (1.3)	0 (0)	0 (0)	0 (0)	0 (0)	1 (1.1)	0 (0)	0 (0)	0 (0)	1 (1.5)	0 (0)	1 (1.5)
Non-extended spectrum cephalosporins												

CZ[a,b]	15 (20.0)	10 (16.1)	9 (9.3)	41 (46.1)	52 (56.5)	39 (43.8)	15 (25.0)	0 (0)	10 (16.1)	15 (22.7)	21 (30.9)	31 (46.3)
CXM[a,b]	15 (20.0)	9 (14.5)	9 (9.3)	43 (48.3)	51 (55.4)	37 (41.6)	14 (23.3)	0 (0)	10 (16.1)	14 (21.2)	19 (27.9)	28 (41.8)
CMZ[a]	2 (2.7)	5 (8.1)	4 (4.1)	18 (20.2)	31 (33.7)	22 (24.7)	1 (1.7)	0 (0)	2 (3.2)	2 (3.0)	6 (8.8)	6 (9.0)
Extended-spectrum cephalosporins												
CRO[a,b]	16 (21.3)	9 (14.5)	9 (9.3)	40 (44.9)	50 (54.3)	37 (41.6)	14 (23.3)	0 (0)	9 (14.5)	15 (22.7)	19 (27.9)	31 (46.3)
CAZ[a]	12 (16.0)	10 (16.1)	8 (8.2)	32 (36.0)	47 (51.1)	31 (34.8)	10 (16.7)	0 (0)	6 (9.7)	14 (21.2)	13 (19.1)	16 (23.9)
FEP[a]	9 (12.0)	4 (6.5)	4 (4.1)	18 (20.2)	18 (19.6)	19 (21.3)	10 (16.7)	0 (0)	5 (8.1)	11 (16.7)	10 (14.7)	15 (22.4)
Cephamycins												
FOX[a]	8 (10.7)	7 (11.3)	8 (8.2)	27 (30.3)	44 (47.8)	30 (33.7)	3 (4.9)	0 (0)	4 (6.5)	5 (7.6)	9 (13.2)	9 (13.4)
Fluoroquinolones												
CIP[a,b]	20 (26.7)	13 (21.0)	25 (25.8)	42 (47.2)	60 (65.2)	51 (57.3)	20 (33.3)	4 (23.5)	20 (32.3)	26 (39.4)	40 (58.8)	39 (58.2)
LVX[a,b]	15 (20.0)	9 (14.5)	18 (18.6)	34 (38.2)	50 (54.3)	43 (48.3)	16 (26.7)	1 (5.9)	13 (21.0)	18 (27.3)	33 (48.5)	36 (53.7)
Tetracyclines												
TE	46 (61.3)	36 (58.1)	52 (53.6)	54 (60.7)	54 (58.7)	52 (58.4)	29 (48.3)	8 (47.1)	27 (43.5)	36 (54.5)	35 (51.5)	33 (49.3)
Glycylcyclines												
TIG	0 (0)	0 (0)	0 (0)	0 (0)	0 (0)	0 (0)	0 (0)	0 (0)	0 (0)	0 (0)	0 (0)	0 (0)
Folate pathway inhibitors												
SXT[a]	41 (54.7)	30 (48.4)	36 (37.1)	47 (52.8)	54 (58.7)	54 (60.7)	32 (53.3)	6 (35.3)	24 (38.7)	26 (39.4)	34 (50.0)	24 (35.8)
Drug-resistance[a]												
MDR	39 (52.0)	29 (46.8)	36 (37.1)	54 (60.7)	64 (70.0)	57 (64.0)	36 (60.0)	7 (41.2)	29 (46.8)	36 (54.5)	39 (57.4)	40 (59.7)
XDR	1 (1.3)	2 (3.2)	2 (2.1)	5 (5.6)	11 (12.0)	5 (5.6)	0 (0)	0 (0)	2 (3.2)	1 (1.5)	5 (7.4)	4 (6.0)

[a]The distribution of antimicrobial resistance of *E. coli* isolated from different age groups in 2009-2010 showed a significant difference ($p < 0.05$).

[b]The distribution of antimicrobial resistance of *E. coli* isolated from different age groups in 2020 showed a significant difference ($p < 0.05$).

Abbreviations: AM, ampicillin; AMC, amoxicillin; AN, amikacin; CAZ ceftazidime, CIP, ciprofloxacin; CMZ, cefmetazole; CRO, ceftriaxone; CXM, cefuroxime; CZ, cefazolin; ETP, ertapenem; FEP, cefepime; FOX, cefoxitin; GM, gentamicin; LVX, levofloxacin; IPM, imipenem; MEM meropenem; SAM, ampicillin/sulbactam; SXT, sulfamethoxazole/trimethoprim; TE, tetracycline; TIG, tigecycline; TZP, piperacillin/tazobactam; I, intermediate resistant; R, resistant; MDR, multidrug-resistant; XDR, extensively drug-resistant.

Table S2. The distribution of virulence factors in *E. coli* in different age groups in 2009-2010 and 2020.

Virulence factor genes	Age group (years old) in 2009-2010						Age group (years old) in 2020					
	≤3 (n=75)	4-20 (n=62)	21-40 (n=97)	41-60 (n=89)	61-80 (n=92)	>80 (n=89)	≤3 (n=60)	4-20 (n=17)	21-40 (n=62)	41-60 (n=66)	61-80 (n=68)	>80 (n=67)
papGI	0 (0)	0 (0)	0 (0)	0 (0)	0 (0)	0 (0)	0 (0)	0 (0)	0 (0)	0 (0)	1 (1.5)	0 (0)
papGII[a,b]	35 (46.7)	17 (27.4)	34 (35.1)	22 (24.7)	17 (18.5)	19 (21.3)	30 (50.0)	5 (29.4)	13 (30.0)	11 (16.7)	8 (11.8)	10 (14.9)
papGIII	17 (22.7)	6 (9.7)	23 (23.7)	16 (18.0)	14 (15.2)	9 (10.1)	4 (6.7)	1 (5.9)	8 (12.9)	13 (19.7)	7 (10.3)	8 (11.9)
sfa	10 (13.3)	5 (8.1)	11 (11.3)	6 (6.7)	2 (2.2)	9 (10.1)	9 (15.0)	0 (0)	9 (14.5)	11 (16.7)	6 (8.8)	8 (11.9)
foc[a]	8 (10.7)	13 (21.0)	12 (12.4)	9 (10.1)	5 (5.4)	6 (6.7)	12 (20.0)	1 (5.9)	10 (16.1)	7 (10.6)	5 (7.4)	5 (7.5)
cnf1[a,b]	26 (34.7)	17 (27.4)	28 (28.9)	15 (16.9)	10 (10.9)	16 (18.0)	23 (38.3)	6 (35.3)	21 (33.9)	21 (31.8)	10 (14.7)	11 (16.4)
aer[b]	56 (74.7)	35 (56.5)	54 (55.7)	56 (62.9)	61 (66.3)	62 (69.7)	47 (78.3)	2 (11.8)	28 (45.2)	35 (53.0)	37 (54.4)	40 (59.7)
usp[a,b]	56 (74.7)	47 (75.8)	68 (70.1)	52 (58.4)	46 (50.0)	49 (55.1)	43 (71.7)	5 (29.4)	32 (51.6)	44 (66.7)	37 (54.4)	39 (58.2)
iha	37 (49.3)	22 (35.5)	28 (28.9)	27 (30.3)	30 (32.6)	36 (40.4)	24 (40.0)	4 (23.5)	15 (24.2)	20 (30.3)	21 (30.9)	27 (40.3)
ompT[a,b]	65 (86.7)	56 (90.3)	84 (86.6)	64 (71.9)	63 (68.5)	68 (76.4)	50 (83.3)	5 (29.4)	44 (71.0)	52 (78.8)	48 (70.6)	54 (80.6)
afa[b]	39 (52.0)	36 (58.1)	44 (45.4)	58 (65.2)	57 (62.0)	50 (56.2)	45 (75.0)	2 (11.8)	39 (62.9)	47 (71.2)	44 (64.7)	40 (59.7)
iRONE[b]	31 (41.3)	31 (50.0)	45 (46.4)	34 (38.2)	33 (35.9)	29 (32.6)	24 (40.0)	6 (35.3)	30 (48.4)	26 (39.4)	16 (23.5)	19 (28.4)
fimH[a]	74 (98.7)	62 (100)	96 (99.0)	89 (100)	85 (92.4)	84 (94.4)	56 (93.3)	14 (82.4)	52 (83.9)	61 (92.4)	59 (86.8)	61 (91.0)
hly[a]	33 (44.0)	15 (24.2)	28 (28.9)	16 (18.0)	16 (17.4)	17 (19.1)	25 (41.7)	9 (52.9)	20 (32.3)	18 (27.3)	15 (22.1)	18 (26.9)
sat[a]	36 (48.0)	16 (25.8)	25 (25.8)	24 (27.0)	30 (32.6)	34 (38.2)	28 (46.7)	2 (11.8)	25 (40.3)	22 (33.3)	33 (48.5)	29 (43.3)
K1[a]	18 (24.0)	27 (43.5)	37 (38.1)	16 (18.0)	11 (12.0)	17 (19.1)	11 (18.3)	5 (29.4)	15 (24.2)	13 (19.7)	14 (20.6)	17 (25.4)

[a]The distribution of virulence factors in *E. coli* isolated from different age groups in 2009-2010 showed a significant difference ($p < 0.05$).

[b]The distribution of virulence factors in *E. coli* isolated from different age groups in 2020 showed a significant difference ($p < 0.05$).

Table S3. Oligonucleotide primers used in this study

Primer	Sequence (5′-3′)	Reference
**Virulence factors detection**		
afa-F	CGGCTTTTCTGCTGAACTGGCAGGC	[1]
afa-R	CCGTCAGCCCCCACGGCAGACC	
iroN-F	AAGTCAAAGCAGGGGTTGCCCG	[2]
iroN-R	GACGCCGACATTAAGACGCAG	
usp-1	CGGCTCTTACATCGGTGCGTTG	[3]
usp-2	GACATATCCAGCCAGCGAGTTC	
foc-F	GGTGGAACCGCAGAAAATAC	[4]
foc-R	GAACTGTTGGGGAAAGAGTG	
sfa-F	GGATGTTTCTTTGGGTAATCTG	[4]
sfa-R	CATTCCCTGTATTCGCATAG	
iha-F	CTGGCGGAGGCTCTGAGATCA	[2]
iha-R	TCCTTAAGCTCCCGCGGCTGA	
ompT-F	ATCTAGCCGAAGAAGGAGGC	[5]
ompT-R	CCCGGGTCATAGTGTTCATC	
papGI-F	TCGTGCTCAGGTCCGGAATTT	[6]
papGI-R	TGGCATCCCCCAACATTATCG	
papGII-F	GGGATGAGCGGGCCTTTGAT	[6]
papGII-R	CGGGCCCCCAAGTAACTCG	
papGIII-1	GGCCTGCAATGGATTTACCTGG	[6]
papGIII-2	CCACCAAATGACCATGCCAGAC	
neuA-F	ATGATTACTCGACACTGTC	[7]
neuA-R	AACAATCTCCGCTATTTCG	
iutA-F	GGCTGGACATCATGGGAACTGG	[2]
iutA-R	CGTCGGGAACGGGTAGAATCG	
cnf1-F	AGACGATTTTATCAATGACCCC	[8]
cnf1-R	CAAAAGACAGACCAAGCAATAC	
sat-F	ACGGTCAGGGATTTACATTT	[9]
sat-R	GCTATTTGGCTGTTATGTGC	
fimH-F	CATTCGCCTGTAAAACCGCC	[10]
fimH-R	ATAACACGCCGCCATAAGCC	
hlyA-F	AGCAATGCAGATGCAGATAC	[8]
hlyA-R	AATGGACAGGAATGAGAGGG	
**Phylogenetic grouping**		[11]
chuA.1b	GACGAACCAACGGTCAGGAT	

chuA.2	TGCCGCCAGTACCAAAGACA
yjaA.1b	TGAAGTGTCAGGAGACGCTG
yjaA.2b	ATGGAGAATGCGTTCCTCAAC
TspE4C2.1	CACTATTCGTAAGGTCATCC
TspE4C2.2	AGTTTATCGCTGCGGGTCGC
AceK.f	AACGCTATTCGCCAGCTTGC
ArpA1.r	TCTCCCCATACCGTACGCTA
ArpAgpE.f	GATTCCATCTTGTCAAAATATGCC
ArpAgpE.r	GAAAAGAAAAAGAATTCCCAA
trpAgpC.1	AGTTTTATGCCCAGTGCGAG
trpAgpC.2	TCTGCGCCGGTCACGCCC
aesI-F	CCTCTACTCACCCAAAAGTC
aesI-R	ATCACGTAACCACAACGCAC

References:

1. Tarchouna, M.; Ferjani, A.; Ben-Selma, W.; Boukadida, J. Distribution of uropathogenic virulence genes in Escherichia coli isolated from patients with urinary tract infection. *Int J Infect Dis* **2013**, *17*, e450-453, doi:10.1016/j.ijid.2013.01.025.
2. Guiral, E.; Bosch, J.; Vila, J.; Soto, S.M. Prevalence of Escherichia coli among samples collected from the genital tract in pregnant and nonpregnant women: relationship with virulence. *FEMS Microbiol Lett* **2011**, *314*, 170-173, doi:10.1111/j.1574-6968.2010.02160.x.
3. Nipic, D.; Podlesek, Z.; Budic, M.; Crnigoj, M.; Zgur-Bertok, D. Escherichia coli uropathogenic-specific protein, Usp, is a bacteriocin-like genotoxin. *J Infect Dis* **2013**, *208*, 1545-1552, doi:10.1093/infdis/jit480.
4. Mitsumori, K.; Terai, A.; Yamamoto, S.; Yoshida, O. Identification of S, F1C and three PapG fimbrial adhesins in uropathogenic Escherichia coli by polymerase chain reaction. *FEMS Immunol Med Microbiol* **1998**, *21*, 261-268, doi:10.1111/j.1574-695X.1998.tb01173.x.
5. Paniagua-Contreras, G.L.; Hernandez-Jaimes, T.; Monroy-Perez, E.; Vaca-Paniagua, F.; Diaz-Velasquez, C.; Uribe-Garcia, A.; Vaca, S. Comprehensive expression analysis of pathogenicity genes in uropathogenic Escherichia coli strains. *Microb Pathog* **2017**, *103*, 1-7, doi:10.1016/j.micpath.2016.12.008.
6. Johnson, J.R.; Brown, J.J. A novel multiply primed polymerase chain reaction assay for identification of variant papG genes encoding the Gal(alpha 1-4)Gal-binding PapG adhesins of Escherichia coli. *J Infect Dis* **1996**, *173*, 920-926, doi:10.1093/infdis/173.4.920.

7. Wang, M.C.; Lin, W.H.; Tseng, C.C.; Wu, A.B.; Teng, C.H.; Yan, J.J.; Wu, J.J. Role of K1 capsule antigen in cirrhotic patients with Escherichia coli spontaneous bacterial peritonitis in southern Taiwan. *Eur J Clin Microbiol Infect Dis* **2013**, *32*, 407-412, doi:10.1007/s10096-012-1757-9.
8. Tseng, C.C.; Wu, J.J.; Liu, H.L.; Sung, J.M.; Huang, J.J. Roles of host and bacterial virulence factors in the development of upper urinary tract infection caused by Escherichia coli. *Am J Kidney Dis* **2002**, *39*, 744-752, doi:10.1053/ajkd.2002.32992.
9. Mao, B.H.; Chang, Y.F.; Scaria, J.; Chang, C.C.; Chou, L.W.; Tien, N.; Wu, J.J.; Tseng, C.C.; Wang, M.C.; Chang, C.C.; et al. Identification of Escherichia coli genes associated with urinary tract infections. *J Clin Microbiol* **2012**, *50*, 449-456, doi:10.1128/JCM.00640-11.
10. Edelstein, M.; Pimkin, M.; Palagin, I.; Edelstein, I.; Stratchounski, L. Prevalence and molecular epidemiology of CTX-M extended-spectrum beta-lactamase-producing Escherichia coli and Klebsiella pneumoniae in Russian hospitals. *Antimicrob Agents Chemother* **2003**, *47*, 3724-3732, doi:10.1128/AAC.47.12.3724-3732.2003.
11. Clermont, O.; Christenson, J.K.; Denamur, E.; Gordon, D.M. The Clermont Escherichia coli phylo-typing method revisited: improvement of specificity and detection of new phylo-groups. *Environ Microbiol Rep* **2013**, *5*, 58-65, doi:10.1111/1758-2229.12019.
